# Emergence and Targeting of Acquired and Hereditary Resistance to Multikinase RET Inhibition in Patients With RET-Altered Cancer

**DOI:** 10.1200/PO.19.00189

**Published:** 2019-09-11

**Authors:** Lori J. Wirth, Takashi Kohno, Hibiki Udagawa, Shingo Matsumoto, Genichiro Ishii, Kevin Ebata, Brian B. Tuch, Edward Y. Zhu, Michele Nguyen, Steve Smith, Lauren M. Hanson, Michael R. Burkard, LouAnn Cable, James F. Blake, Kevin R. Condroski, Barbara J. Brandhuber, Steve Andrews, S. Michael Rothenberg, Koichi Goto

**Affiliations:** ^1^ Massachusetts General Hospital Cancer Center, Boston, MA; ^2^ National Cancer Research Institute, Tokyo, Japan; ^3^ National Cancer Center Hospital East, Kashiwa, Japan; ^4^ Loxo Oncology, Stamford, CT; ^5^ Array BioPharma, Boulder, CO

## INTRODUCTION

The RET receptor tyrosine kinase is oncogenically activated by *RET* gene fusions in 1% to 2% of non–small-cell lung cancers (NSCLC) and by *RET* gene mutations in most medullary thyroid cancers (MTC).^1^ Although multikinase inhibitors (MKIs) with nonspecific anti-RET activity are approved for the treatment of MTC irrespective of *RET* mutation status, their investigational use for patients with *RET* fusion-positive NSCLC has been limited by substantial off-target adverse effects that lead to dose reductions and inadequate RET-specific inhibition.^2-7^ As a result, tumor responses to MKIs have been infrequent and short lived, and a comprehensive molecular understanding of MKI response and resistance is lacking.

In contrast to the MKIs, LOXO-292 is a highly selective, small molecule RET tyrosine kinase inhibitor (TKI) with nanomolar potency against diverse RET alterations, favorable pharmacokinetic (PK) properties, and significant CNS penetration.^8^ In an ongoing, phase I/II study of LOXO-292 (ClinicalTrials.gov identifier: NCT03157128), confirmed partial responses (PRs) were achieved in most *RET*-altered patients across different tumor histologies and diverse *RET* gene fusions and mutations. Moreover, activity has been seen after acquired resistance to systemic therapies, including MKIs, and in intracranial disease.^9-11^ Responses have been generally long lasting with the median duration of response not yet reached, and the safety profile has been tolerable, consistent with preclinical high selectivity.

LOXO-292 was designed to overcome mutations in the RET V804 gatekeeper residue shown previously to cause preclinically acquired resistance to anti-RET MKIs.^8,12^ Recently, we reported the emergence of a RET valine-to-methionine (V804M) mutation in circulating tumor DNA from a patient with *RET*-mutant, sporadic MTC treated previously with multiple MKIs, and the ability of LOXO-292 to induce a durable tumor response in this patient.^13^ Two other reports described the emergence of RET kinase domain mutations (RET V804M and RET S904F) each in a single patient with RET fusion-positive NSCLC during treatment with the anti-RET MKI vandetanib.^14,15^ Their overall frequency and clinical actionability are not clear.

Here, we describe the ability of LOXO-292 to overcome acquired RET kinase domain mutations preclinically and in two patients with *RET*-altered cancers, one with acquired gatekeeper mutation resistance to an anti-RET MKI and the second with a germline gatekeeper mutation in the setting of multiple endocrine neoplasia 2A.

## CASE REPORT

### Case 1: KIF5B-RET fusion-positive NSCLC.

**FIG 1.**
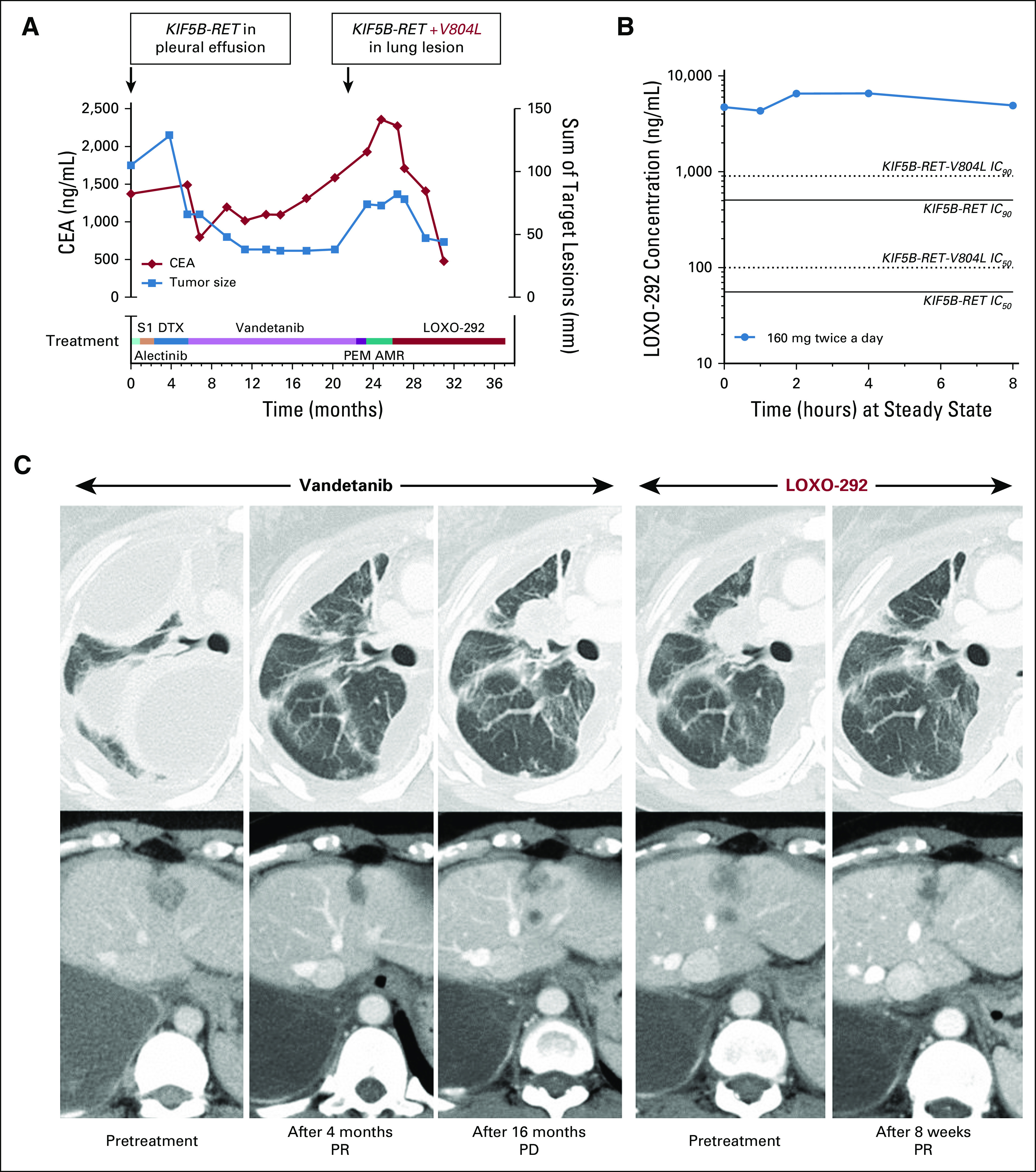
Clinical activity of LOXO-292 in a patient with *RET* fusion-positive non–small-cell lung cancer after acquired resistance to vandetanib. (A) Various treatments the patient received for metastatic, KIF5B-RET fusion-positive non–small-cell lung cancer are shown, together with the levels of carcinoembryonic antigen (CEA; red diamonds) and the sum of target lesions by Response Evaluation Criteria in Solid Tumors (RECIST) 1.1 (blue squares) over time during treatment. The *RET* molecular profiles of tumor specimens obtained before and during vandetanib treatment are shown in boxes. (B) Pharmacokinetic analysis at steady state after treatment with LOXO-292 at a dose of 160 mg twice per day for 8 days. The estimated levels of RET target inhibition at the 50% inhibitory concentration (IC_50_) and 90% inhibitory concentration (IC_90_) for KIF5B-RET and KIF5B-RET-V804L were modeled using actual patient human pharmacokinetic parameters (Data Supplement). (C) Computed tomographic images of the patient’s metastatic lung and liver disease before and at the indicated times after initiating treatment with vandetanib (left three pairs of panels) or LOXO-292 (right two pairs of panels). AMR, ambirubicin; DTX, docetaxel; PD, progressive disease; PEM, pemetrexed; PR, partial response; S1, tegafur plus gimeracil plus oteracil.

A 42-year-old woman with NSCLC had received nine prior systemic therapy regimens, including approved chemotherapy, immunotherapy, and investigational TKIs, before the identification of a *KIF5B-RET* fusion in a sample from a recurrent pleural fusion (Fig 1A, Data Supplement). She received alectinib and additional chemotherapy and then vandetanib (Fig 1A).^6^ She experienced a confirmed PR by Response Evaluation Criteria in Solid Tumors (RECIST) 1.1 with vandetanib treatment, with improvement in multiple lung and pleural-based lesions. She progressed after 13 months of treatment (Figs 1A and 1C). Targeted *RET* gene sequencing of a biopsy specimen from a progressing right lung lesion identified a RET valine-to-leucine (V804L) gatekeeper mutation absent from a pretreatment tumor sample (Fig 1A, Data Supplement). V804L allele frequency in the progression sample was 15% by next-generation sequencing (120 of 799 reads) compared with 0% in the pretreatment sample (0 of 847 reads; Data Supplement).

The patient received additional systemic chemotherapy with pemetrexed and amrubicin but experienced continued disease progression. After providing written informed consent, she started treatment with LOXO-292 in the phase I portion of the phase I/II clinical trial at the recommended phase II dose of 160 mg twice a day. Real-time PK analysis revealed significant (more than 90% inhibitory concentration [IC_90_]) calculated KIF5B-RET and KIF5B-RET-V804L target coverage at steady state (Fig 1B). Consistent with this, she experienced a rapid clinical and biochemical response to LOXO-292, with decreased shortness of breath and reduction in serum carcinoembryonic antigen (Fig 1A). Repeat imaging after 8 weeks of treatment demonstrated a 48% decrease in measurable tumor burden by RECIST 1.1 that was maintained after 16 weeks, indicating a confirmed PR (Figs 1A and 1C). The patient has received LOXO-292 for more than 11 months, is still receiving treatment, and is tolerating therapy well, without dose interruption, and with all treatment-emergent adverse events grade 1 or 2.

### Case 2: Hereditary RET V804M MTC.

**FIG 2.**
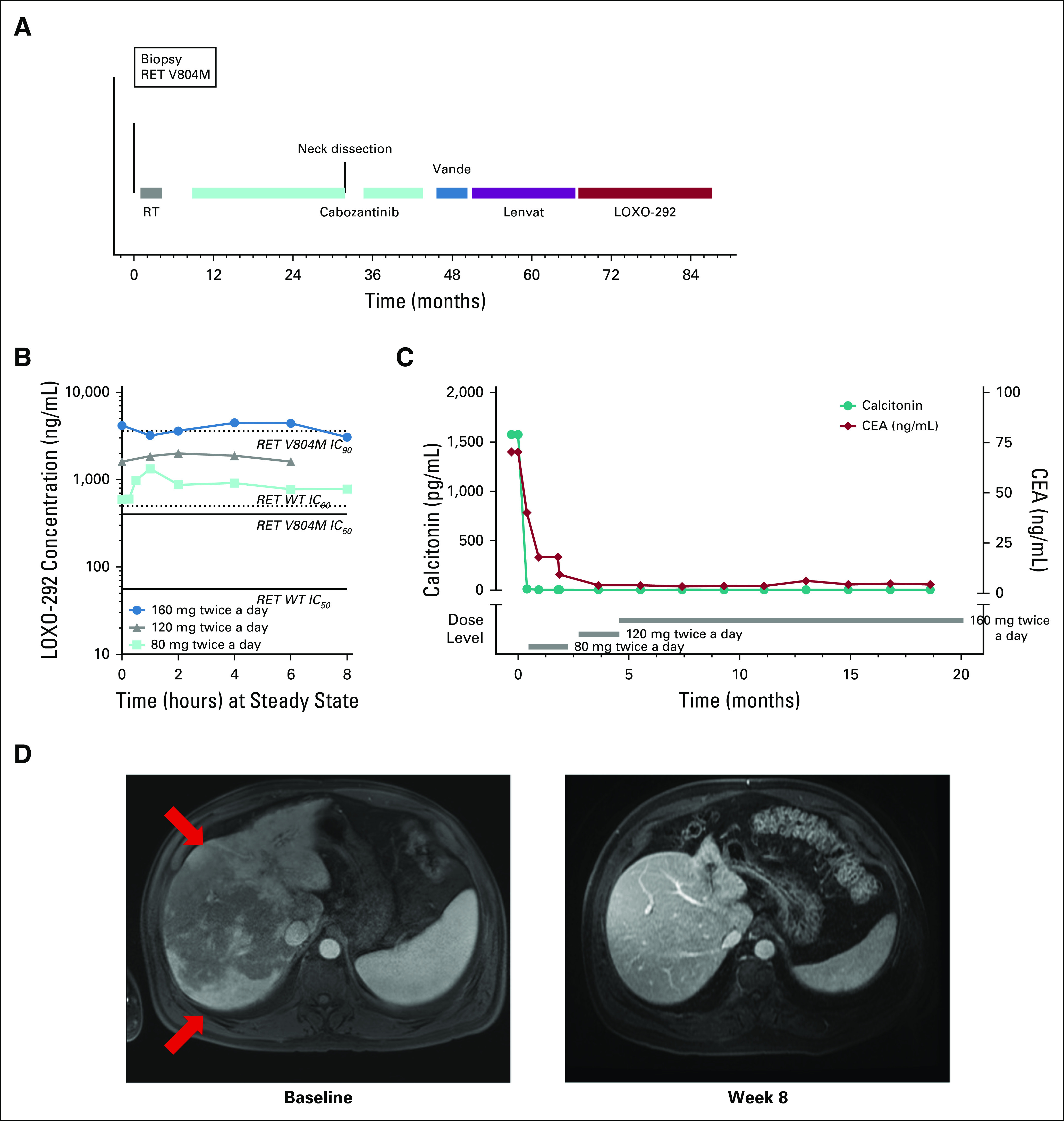
Clinical activity of LOXO-292 in a patient with *RET*-mutant medullary thyroid cancer with a germline V804M gatekeeper mutation. (A) The treatments the patient received for metastatic, RET V804M-mutant medullary thyroid cancers are shown. (B) Pharmacokinetic analysis at steady state after 8 days of treatment with LOXO-292 at a dose of 80 mg, 120 mg, and 160 mg twice a day. The estimated levels of RET target inhibition at the 50% inhibitory concentration (IC_50_) and 90% inhibitory concentration (IC_90_) for wild-type (WT) RET and RET V804M were modeled as in Fig 1B. (C) Levels of calcitonin (green circles) and carcinoembryonic antigen (CEA; red diamonds) over time during treatment. (D) Computed tomographic images of the patient’s metastatic liver disease before and after 8 weeks of treatment with LOXO-292. The red arrows indicate diffuse infiltration of the liver by tumor. Lenvat, lenvatinib; RT, radiation treatment; Vande, vandetanib.

A 50-year-old man with hereditary RET V804M multiple endocrine neoplasia 2A and MTC developed primary disease progression despite previous treatment with three anti-RET MKIs (Fig 2A). Before treatment with LOXO-292, he was highly symptomatic, with abdominal pain from tumor infiltration of the liver and severe tumor-related diarrhea. LOXO-292 was initiated at a dose of 80 mg twice a day during the phase I portion of the clinical trial. The patient provided written informed consent before enrollment. PK analysis indicated more than 50% inhibitory concentration calculated RET V804M target coverage at this dose (Fig 2B). Consistent with this, serum carcinoembryonic antigen and calcitonin levels decreased rapidly, together with resolution of diarrhea, abdominal distension, and abdominal pain. Repeat imaging after 8 weeks of treatment indicated a complete response by RECIST 1.1, which was confirmed after 12 weeks of treatment, with complete resolution of target mediastinal lymph node lesions and nontarget liver metastases and normalization of serum tumor markers (Figs 2C and 2D). The patient’s dose was increased to 160 mg twice a day as allowed by the protocol, with dose-proportional increases in exposure and continuous more than IC_90_ RET V804M target coverage (Fig 2B), continued complete response, and continued normalization of tumor markers (Fig 2C). The patient experienced three adverse events: transient grade 3 altered mental status (sedation), judged by the investigator to be related to concomitant treatment with anxiolytic medications and unrelated to LOXO-292; grade 1 constipation; and grade 2 nausea. He remains in complete response and is still receiving treatment at more than 20 months after the start of LOXO-292.

### LOXO-292 but not anti-RET MKIs maintain inhibitory activity against RET gatekeeper mutations preclinically.

**FIG 3.**
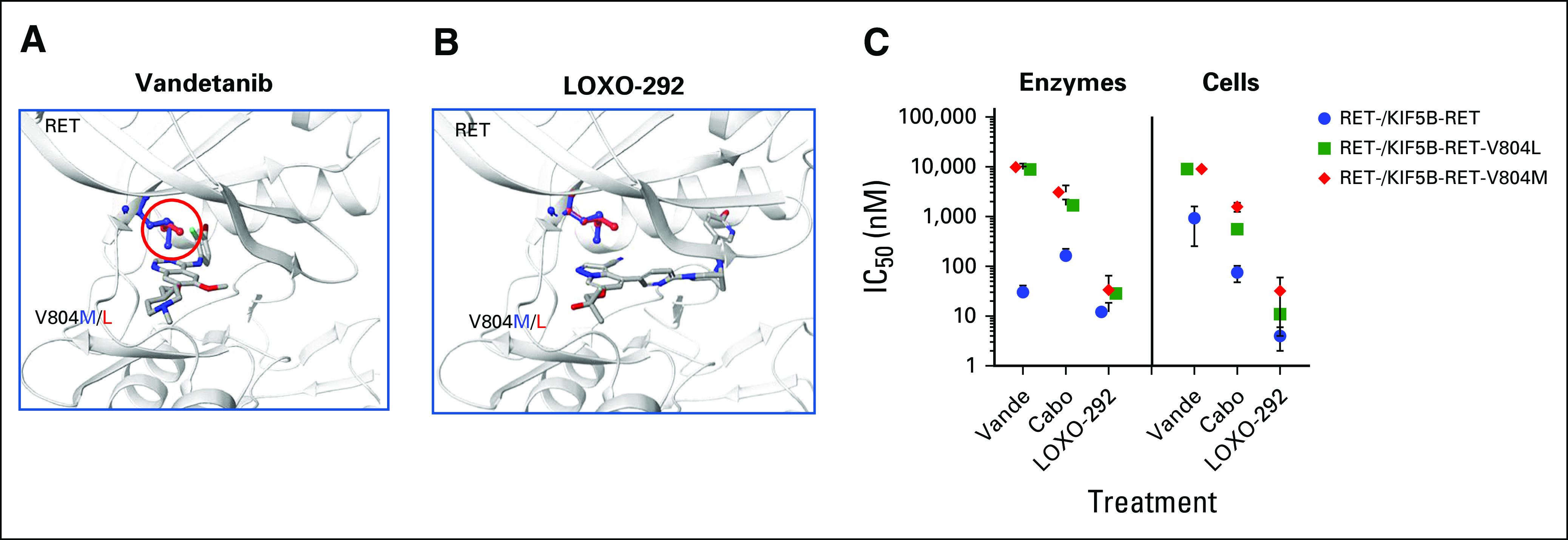
RET inhibitor binding to RET resistance mutations. (A) Structural models showing steric interactions (circled red) between vandetanib (Vande) and gatekeeper (V804L and V804M) resistance mutations in RET. (B) Similar model indicating that LOXO-292 can accommodate V804L and V804M without significant steric hindrance. (C) The 50% inhibitory concentration (IC_50_) values for Vande, cabozantinib (Cabo), and LOXO-292 in (left) purified kinase assays at 1mM ATP and (right) cell-based assays of RET autophosphorylation are shown as mean ± SEM of 5 to 145 (enzyme) and 2 to 55 (cell) replicates.

We used structural modeling to understand how RET kinase domain mutations might affect anti-RET TKI binding activity. V804L and V804M substitutions in the gatekeeper residue of RET are predicted to introduce steric clashes between the leucine and methionine side chains and the 4-bromo-2-fluorophenyl group of the anti-RET MKI vandetanib (Fig 3A). In contrast to MKIs, LOXO-292 is predicted to better accommodate the bulky leucine and methionine side chains in the gatekeeper residues without any steric interactions (Fig 3B).

To determine the impact of RET kinase domain resistance mutations on inhibitor activity, LOXO-292, vandetanib, and cabozantinib were tested in vitro against the purified wild-type and mutant RET kinase domains (Fig 3C). At physiologic (1 mM) ATP concentration, LOXO-292 had potent inhibitory activity against wild-type RET, RET V804L, and RET V804M kinases. In contrast, the anti-RET MKIs, vandetanib and cabozantinib, displayed significantly reduced activity against RET V804L/M compared with wild-type RET. These findings were confirmed in cell-based assays of RET autophosphorylation (Fig 3C). These data demonstrate that LOXO-292, but not the MKIs vandetanib or cabozantinib, maintain potent inhibitory activity against RET gatekeeper mutations.

## DISCUSSION

Selective TKIs targeting actionable kinase alterations have transformed the treatment landscape for several human cancers. Although acquired resistance to TKIs is universal, a detailed molecular understanding of resistance has led to the development of next-generation TKIs that have extended disease control and clinical benefit in resistant patients.^16,17^ In several instances, initial treatment with the next-generation inhibitor has led to more durable treatment responses than treatment with the first-generation TKI and has become standard upfront systemic therapy.^18,19^

LOXO-292 combines features of both first- and next-generation TKIs used to treat other single kinase-driven cancers. It is highly potent against and selective for diverse founder activating RET alterations in human cancers, and in preclinical experiments, it overcomes RET V804 gatekeeper mutations, which have been identified in rare patients with acquired resistance to anti-RET MKIs. Gatekeeper mutations in other addicting kinases cause acquired resistance to other first-generation TKIs (eg, imatinib, erlotinib, and crizotinib), but this resistance may be overcome by next-generation inhibitors (eg, ponatinib, osimertinib, and alectinib).^20-22^

To our knowledge, case 1 is the second patient with NSCLC to be described as having an acquired RET gatekeeper mutation after prior anti-RET MKI treatment and the first to be treated successfully with RET-targeted therapy.^14^ To our knowledge, this is also the first published report of a patient with hereditary RET V804M-mutant MTC treated successfully with RET-targeted therapy. For both patients, integration of preclinical RET target inhibitory activity with the actual exposures safely achieved at the recommended phase II dose of 160 mg twice a day allowed more than IC_90_ calculated target coverage of both wild-type and gatekeeper-mutated RET in patients (Figs 1B and 2B). Consistent with this, sequential treatment with LOXO-292 after the prior MKIs has significantly extended the period of durable clinical and biochemical response and disease control. Additional follow-up will be required to determine whether activity against RET gatekeeper mutations translates into more durable treatment responses when LOXO-292 is used upfront rather than sequentially after MKIs at the time of resistance.

In conclusion, the highly selective anti-RET TKI LOXO-292 led to durable tumor responses in two patients with RET V804 gatekeeper mutations, one acquired and one germline. These results illustrate the importance of anticipating mechanisms of acquired resistance when developing new targeted therapies and underscore the potential of selective RET inhibition with LOXO-292 to treat patients with *RET*-altered cancer exposed previously to anti-RET MKIs.

## Data Availability

The following represents disclosure information provided by authors of this manuscript. All relationships are considered compensated. Relationships are self-held unless noted. I = Immediate Family Member, Inst = My Institution. Relationships may not relate to the subject matter of this manuscript. For more information about ASCO's conflict of interest policy, please refer to www.asco.org/rwc or ascopubs.org/po/author-center. **Consulting or Advisory Role:** Merck, Loxo Oncology, Blueprint Medicines, Eisai, Amgen, Novartis, Ayala Pharmaceuticals, CUE Biopharma, Genentech, Iovance Biotherapeutics, Rakuten Medical **Other Relationship:** Iovance Biotherapeutics **Consulting or Advisory Role:** Loxo Oncology **Research Funding:** Daiichi Sankyo, Sysmex **Honoraria:** AstraZeneca, Chugai Pharmaceutical, Bristol-Myers Squibb, Ono Pharmaceutical, MSD, Taiho Pharmaceutical, Amco, AbbVie, Boehringer Ingelheim, Daiichi Sankyo **Consulting or Advisory Role:** AbbVie, Boehringer Ingelheim **Research Funding:** MSD, AbbVie, Daiichi Sankyo, Amgen **Honoraria:** Pfizer, Novartis, Chugai Pharma, AstraZeneca **Research Funding:** Chugai Pharma (Inst), Novartis (Inst), Lilly (Inst), Merck Serono (Inst), Merck Sharp & Dohme (Inst) **Employment:** Loxo Oncology **Stock and Other Ownership Interests:** Loxo Oncology **Travel, Accommodations, Expenses:** Loxo Oncology **Employment:** Loxo Oncology **Stock and Other Ownership Interests:** Loxo Oncology **Consulting or Advisory Role:** Kezar Life Sciences **Patents, Royalties, Other Intellectual Property:** Biomarkers for NTRK inhibition; biomarkers for proteasome inhibition **Employment:** Loxo Oncology **Stock and Other Ownership Interests:** Loxo Oncology **Travel, Accommodations, Expenses:** Loxo Oncology **Employment:** Loxo Oncology **Stock and Other Ownership Interests:** Loxo Oncology **Travel, Accommodations, Expenses:** Loxo Oncology **Consulting or Advisory Role:** Loxo Oncology, various **Patents, Royalties, Other Intellectual Property:** Various patents and applications **Travel, Accommodations, Expenses:** Various **Employment:** Array BioPharma **Stock and Other Ownership Interests:** Array BioPharma **Research Funding:** Array BioPharma **Employment:** Array Biopharma, University of Colorado Hospital (I) **Stock and Other Ownership Interests:** Array BioPharma, Pfizer, Pfizer (I) **Employment:** Array BioPharma **Stock and Other Ownership Interests:** Array BioPharma **Patents, Royalties, Other Intellectual Property:** I am listed as a co-author on approximately 46 patents (Inst) **Employment:** Loxo Oncology **Stock and Other Ownership Interests:** Loxo Oncology **Travel, Accommodations, Expenses:** Loxo Oncology **Employment:** Loxo Oncology **Stock and Other Ownership Interests:** Loxo Oncology, Array BioPharma **Travel, Accommodations, Expenses:** Loxo Oncology **Employment:** Loxo Oncology **Leadership:** Loxo Oncology **Stock and Other Ownership Interests:** Loxo Oncology **Travel, Accommodations, Expenses:** Loxo Oncology **Employment:** Loxo Oncology **Stock and Other Ownership Interests:** Loxo Oncology **Travel, Accommodations, Expenses:** Loxo Oncology **Honoraria:** Bristol-Myers Squibb, AstraZeneca, Pfizer, Chugai Pharmaceutical, Taiho Pharmaceutical, Ono Pharmaceutical, Novartis, Eli Lilly, Boehringer Ingelheim, Quintiles, Merck Serono, Life Technologies, MSD, AbbVie, Riken Genesis, Nippon Kayaku, Takeda Pharmaceuticals, Otsuka Pharmaceutical, SRL Diagnostics **Consulting or Advisory Role:** Otsuka Pharmaceutical **Research Funding:** MSD, AstraZeneca, Taiho Pharmaceutical, Chugai Pharmaceutical, Boehringer Ingelheim, Ono Pharmaceutical, Sumitomo Dainippon, Takeda Pharmaceuticals, Novartis, Daiichi Sankyo, Kyowa Hakko Kirin, Astellas Pharma, Eisai, Eli Lilly, Pfizer, Riken Genesis, Bristol-Myers Squibb, Merck Serono, Ignyta, Life Technologies, Research Triangle Institute d/b/a RTI Health Solutions, Janssen, Xcoo, Loxo Oncology No other potential conflicts of interest were reported.
